# Surgery for Pancoast Tumors in Multimodality Setting: Analysis of Outcomes and Risk Factors

**DOI:** 10.3390/jcm14082758

**Published:** 2025-04-17

**Authors:** Giorgio Cannone, Eleonora Faccioli, Alberto Busetto, Luigi Lione, Giuseppe Maggioni, Samuele Nicotra, Marco Schiavon, Alessandro Rebusso, Giovanni Comacchio, Marco Mammana, Matteo Sepulcri, Giulia Pasello, Fiorella Calabrese, Andrea Dell’Amore, Federico Rea

**Affiliations:** 1 Thoracic Surgery Unit, Department of Cardiac, Thoracic, Vascular Sciences and Public Health, University Hospital of Padova, 35121 Padova, Italy; giorgio.cannone@aopd.veneto.it (G.C.); alberto.busetto@aopd.veneto.it (A.B.); luigi.lione@aopd.veneto.it (L.L.); samuele.nicotra@aopd.veneto.it (S.N.); marco.schiavon@unipd.it (M.S.); alessandro.rebusso@aopd.veneto.it (A.R.); giovannimaria.comacchio@aopd.veneto.it (G.C.); marco.mammana@aopd.veneto.it (M.M.); andrea.dellamore@unipd.it (A.D.);; 2 Pathology Unit, Department of Cardiac, Thoracic, Vascular Sciences and Public Health, University Hospital of Padova, 35121 Padova, Italy; giuseppe.maggioni@phd.unipd.it (G.M.); fiorella.calabrese@unipd.it (F.C.); 3 Radiotherapy Unit, Istituto Oncologico Veneto, Department of Medicine, University Hospital of Padova, 35121 Padova, Italy; matteo.sepulcri@iov.veneto.it; 4 Oncology 2 Unit, Istituto Oncologico Veneto, Department of Surgical Oncological and Gastroenterological Sciences, University Hospital of Padova, 35121 Padova, Italy; giulia.pasello@unipd.it

**Keywords:** Pancoast tumor, clinical outcomes, vascular/vertebral invasion, multimodality treatment, pathological complete response

## Abstract

**Background**: Pancoast tumors are a rare subset of lung cancers that require a multimodal approach (induction chemoradiotherapy and surgery), best performed in highly specialized centers. This study analyzes the outcomes and prognostic factors in patients treated at a high-volume center over an extended period. **Methods**: We retrospectively reviewed 43 patients who underwent surgery for Pancoast tumors, following induction treatment between 2005 and 2023. Survival was estimated using the Kaplan–Meier method, and a Cox proportional hazards model was applied to identify prognostic factors (significance level *p* = 0.05). **Results**: The median patient age was 63 years, with over 90% having a disease at stage III or higher. Induction chemoradiotherapy was administered to 79% of the patients, achieving a pathological complete response (PCR) in 23% of the patients. The median overall survival (OS) was 37 months, with 1–3 and 5-year OS rates of 71%, 52%, and 41%, respectively. The median disease-free survival (DFS) was 38 months, with 1-, 3-, and 5-year DFS rates of 72%, 62%, and 35%, respectively. A pathological complete response (PCR) and vertebral and/or vascular infiltration significantly influenced recurrence and mortality risk. **Conclusions**: Trimodal therapy still offers the best short- and long-term outcomes in patients with Pancoast tumors. Future strategies incorporating tyrosine kinase inhibitors and anti-PD1/PD-L1 may improve outcomes for patients by increasing PCR rates and improving disease control.

## 1. Introduction

Pancoast tumors are a subset of non-small-cell lung cancers (NSCLCs), accounting for less than 5% of all NSCLC cases [1]. They most commonly present in around the sixth decade of life, with a higher prevalence in males. These tumors are located at the apex of the upper lobes, developing in the thoracic inlet and typically involving at least the first rib. Their location is responsible for specific symptoms that result from the possible infiltration of anatomical structures of the thoracic inlet [2,3]. For this reason, according to both the 8th and the new 9th edition of the TNM classification, Pancoast tumors are categorized as at least stage T3, classifying them as locally advanced NSCLCs. Progressing to stage T4 primarily depends on the involvement of the subclavian vessels, vertebrae, or brachial plexus.

This unique localization often leads to so-called Pancoast syndrome, which consists of a triad of symptoms: shoulder and arm pain, wasting of the hand muscles, and ipsilateral Horner syndrome (characterized by ptosis, miosis, and anhidrosis, due to the invasion of the stellate ganglion of the sympathetic chain) [4]. However, a Pancoast tumor can be present even without the development of Pancoast syndrome, which can sometimes make diagnosis challenging. The unique localization of this tumor subset makes surgical management particularly complex and challenging. The thoracic inlet is an anatomical region through which neurovascular structures supplying the arm, as well as the sympathetic chain, pass. It is divided into three compartments: anterior, middle, and posterior. A thorough understanding of the anatomy is essential, as these compartments correspond precisely to the three categories of Pancoast tumors, which may represent distinct disease entities. For this reason, meticulous preoperative planning is crucial to assess resectability, preparing the surgeon for the type of pulmonary resection required, the number of ribs to be resected, and, most importantly, the potential need for vascular and/or vertebral resection and reconstruction. In most cases, conventional chest CT scans provide sufficient imaging for surgical planning. However, in certain situations, particularly when vertebral invasion and/or brachial plexus involvement is suspected, magnetic resonance imaging (MRI) can offer additional clarity in defining the extent of surgical resection needed.

The treatment of Pancoast tumors has evolved over the years. In the past, due to their poor prognosis, these tumors were considered inoperable in most cases, with palliative radiotherapy being the only option, primarily for pain control. Later, the standard of care shifted to neoadjuvant radiotherapy, followed by en bloc resection of the lung, along with the involved surrounding structures. Currently, based on evidence reported from US [5] and Japanese trials [6], the gold standard for treating resectable Pancoast tumors is a multimodal approach, which includes neoadjuvant chemoradiotherapy followed by surgery [7,8]. This strategy has demonstrated superiority over radiotherapy alone, as it provides excellent locoregional disease control, with a higher rate of pathological complete response (PCR). Additionally, it is associated with improved distant disease control, leading to a reduction in distant relapses. In addition, despite promising results in terms of lung cancer, both in adjuvant and neoadjuvant settings, tyrosine kinase inhibitors (TKIs) and immunotherapy are still poorly investigated in the context of Pancoast tumors and experiences in this field are limited [9,10], but they deserve further investigation. This is particularly true because, based on the results of various trials, TKIs and/or immunotherapy are associated with higher local response rates and better control of distant disease. These approaches may lead to improved outcomes even in this subset of lung cancers, which have historically been linked to a poor prognosis, and may represent valid treatment alternatives for patients who are not eligible for surgery due to functional or anatomical reasons.

The aim of our study is to analyze the outcomes and prognostic factors in a cohort of patients submitted to multimodal treatment over an extended period at a highly experienced center.

## 2. Materials and Methods

### 2.1. Study Population

A total of 43 patients affected by Pancoast tumors and submitted to surgery in the context of a multimodality setting at the Thoracic Surgery Unit of the University Hospital of Padua, between 2005 and 2023, were included in this study. Pancoast tumor was defined, despite the histology, as a primary lung neoplasia growing at the apex of the lung and invading the homolateral thoracic wall above the level of the second rib and always invading the first rib, otherwise considered as lung cancer invading the chest wall but not definable as Pancoast tumor and, so, excluded from this study. Furthermore, the patients included in this analysis had a histologically confirmed diagnosis of lung cancer and underwent multimodal neoadjuvant treatment, according to our institutional protocol. Following a multidisciplinary discussion, only those deemed suitable for a multimodal approach, including surgery, based on their performance status and cardiopulmonary function were selected. Patients who experienced disease progression after neoadjuvant therapy were excluded from the analysis.

This study was approved by the Institutional Review Board (IRB) of the University Hospital of Padua (5966/AO/24). Due to the retrospective nature of the study, the need for informed consent from the patient was waived.

### 2.2. Operative Management Strategy

At our Institution, all patients diagnosed with a Pancoast tumor undergo staging involving a total-body CT scan, a whole-body 18F-FDG PET, and magnetic resonance imaging in cases of suspected spinal cord invasion. Resectability is always discussed at the beginning of treatment by a multidisciplinary tumor board composed of thoracic surgeons, oncologists, radiation oncologists, radiologists, pulmonologists, and pathologists. If the PET–CT scan findings suggest N2 or N3 disease, the patient undergoes invasive pathological confirmation of nodal involvement using EBUS-TBNA, mediastinoscopy, or VATS.

Before determining eligibility for multimodality protocols, particular attention is given to the patient’s performance status and overall clinical conditions, with a thorough evaluation of their cardio-respiratory function.

If resectability and operability are confirmed, a trimodality therapy approach based on induction chemoradiation therapy, followed by radical surgery, is adopted.

Until 2017, radiation treatment was delivered using three-dimensional conformal radiotherapy (3D-CRT), with daily portal image-guided radiotherapy (IGRT) and a total prescribed dose of 45 Gy in 25 fractions, combined with concomitant chemotherapy (based on carboplatin and paclitaxel every week for 3–4 cycles). From 2018, advancements in technology enabled the use of intensity-modulated arc therapy (VMAT), allowing a dose escalation to 50.4 Gy in 28 fractions. The introduction of cone-beam computed tomography (CBCT) as a form of IGRT facilitated adaptive replanning for tumor shrinkage or anatomical changes (e.g., atelectasis). Target volumes were defined as the gross tumor volume (GTV), based on CT and PET imaging, expanded isotropically by 5–8 mm to create the clinical target volume (CTV), which was manually adjusted to respect anatomical boundaries. The planning target volume (PTV) was generated by adding a 5 mm margin to the CTV. The planning objective required 98% of the PTV (D98%) to be covered by at least 95% of the prescribed dose, while the near-max dose (D2%) was restricted to no more than 107% of the prescribed dose. The safety profile of the induction treatment is assessed based on the occurrence of adverse events (AEs) and serious adverse events (SAEs).

**Figure 1:**
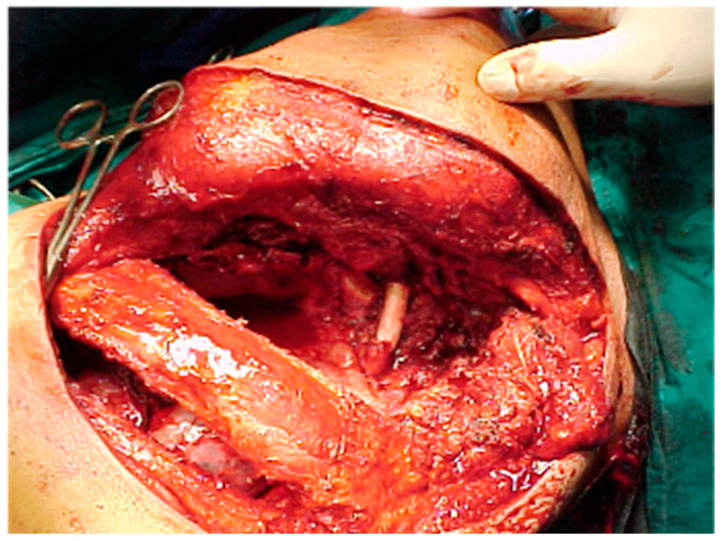
Intraoperative image of an en bloc resection of a lung (lobectomy), the involved chest wall and subclavian artery are reconstructed with a prosthetic replacement, performed through the use of a Shaw–Paulson incision.

Surgery is performed no later than four weeks after the completion of induction treatment to ensure en bloc resection of the lung, along with the involved chest wall, with extension to the vertebrae or vessels in the case of T4 tumors (Figure 1). According to our protocol, in cases where CT imaging suggests invasion of the vertebral body, a chest MRI is always performed. A neurosurgical evaluation is then required, and a neurosurgeon is available in the operating room if vertebral body resection is necessary. Regarding vascular structure invasion, this is initially assessed through preoperative imaging. If confirmed intraoperatively, vascular reconstruction can be performed using either tangential sutures or, in the case of more circumferential invasion, prosthetic conduit reconstruction. For complex vascular bypass procedures, vascular surgeons are available.

The preferred lung resection is always anatomical (lobectomy or segmentectomy), while wedge resection is reserved only for patients with poor respiratory function. A systematic hilo-mediastinal lymphadenectomy is always performed.

The completeness of resection (R0 vs. R+) was assessed by our pathologists in the final pathological report through the analysis of pathological infiltration in the resected specimen, including the lungs, ribs, and vessels. In cases of uncertainty, the extent of resection was determined using frozen section analysis of the tissue margins to confirm clear resection margins. PCR was defined as the total absence (0%) of viable tumor cells using a pathological assessment of the resected specimen (including primary tumor and lymph nodes) [11]. After surgery, our patients undergo periodic follow-up. The follow-up period was defined from the date of surgery to the patient’s last recorded contact, either through an outpatient clinic visit or a phone call for surviving patients, or the date of death for those who did not survive. Disease recurrence was determined based on the date of the first occurrence of either local and/or distant recurrence, measured from the date of surgery.

### 2.3. Statistical Analysis

Descriptive statistics were reported as the median (I–III quartile) for continuous variables and absolute numbers (percentages) for categorical variables. Survival curves were estimated using the Kaplan–Meier method. Univariate logistic regressions were performed to assess the effect of the baseline characteristics on the risk of recurrence and death. A multivariable Cox proportional hazards model was constructed based on the hypothesized clinical relevance. The significance level was set to 5% (*p* = 0.05).

Statistical analysis was conducted using the software Jamovi [The Jamovi project (2022). The Jamovi (version 2.3) computer software was retrieved from https://www.jamovi.org, accessed on 09 December 2024].

## 3. Results

### 3.1. Patients’ Characteristics

The patients’ characteristics are summarized in Table 1. The study population, predominantly composed of smokers or former smokers (93%) and males (77%), had a median age of 63 years. The median Charlson Comorbidity Index (CCI) was 3. Pre-treatment pain was reported by 39 patients (91%), involving only the shoulder in 14 cases (32%), the shoulder and arm in 19 cases (44%), and the shoulder, arm, and hand in 6 cases (14%). Additionally, six patients (14%) presented with Claude Bernard-Horner syndrome prior to treatment. The Pancoast tumor was primarily localized in the right hemithorax (56%). In 30 patients (70%), the pre-treatment diagnostic procedure most used was a CT-guided biopsy, while a bronchoscopy was conducted in 10 cases (30%). According to the 8th edition of the TNM staging system, most patients were clinically categorized as having stage IIIA (51%) or IIIB (31%) disease. One patient was classified as stage IV due to the occurrence of oligo-metastatic disease, with a single metastasis in the adrenal gland, which was also surgically removed.

### 3.2. Data on Multimodal Treatment

The data on the patients’ multimodal treatment are reported in Table 2. The preoperative histology of the primary lung tumor was predominantly adenocarcinoma, observed in 28 patients (60%), followed by squamous cell carcinoma in 12 patients (28%). The most commonly utilized induction protocol consisted of concurrent chemoradiotherapy, applied in 79% of cases. Treatment toxicity was reported in four patients and was mostly related to grade 1 or grade 2 adverse events (AEs). Grade 3 or > 3 AEs were reported in two and one patients, respectively. However, no interruption of the treatment was required and no delays occurred in regard to surgical resection. 

Regarding the surgical approach, the majority of pulmonary resections were lobectomies (84%), performed predominantly through the use of a Shaw–Paulson posterolateral thoracotomy, which was employed in nearly all cases (42 patients, 98%). Vascular and vertebral involvement were noted in five (12%) and four (9%) patients, respectively. Chest wall resection was undertaken in every case, with the number of resected ribs varying; three ribs were resected in 53% of patients. A radical resection (R0) was achieved in 36 patients (84%). At the final histopathological examination, 10 patients (23%) showed a pathological complete response. Recurrence during follow-up was reported in 20 cases (47%), distributed as 13 (30%) local, 5 (12%) distant, and 5 (2%) local + distant. Ten patients (23%) were alive after a median follow-up of 37 months.

### 3.3. Outcomes Analysis and Prognostic Factors

**Figure 2:**
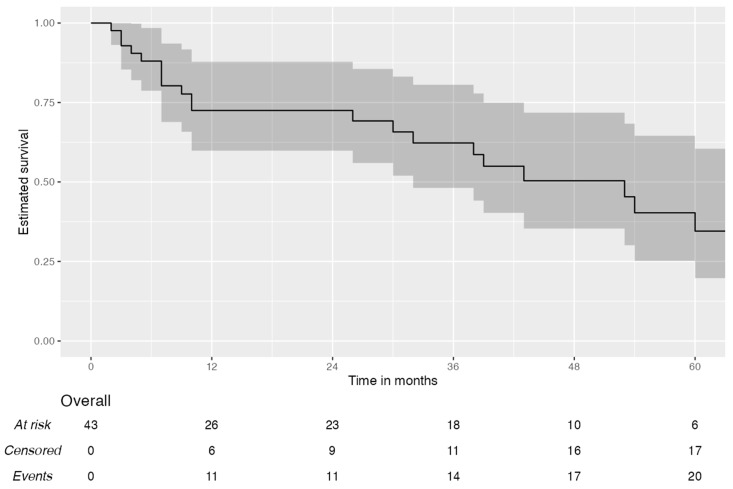
Disease-free survival curve.

**Figure 3:**
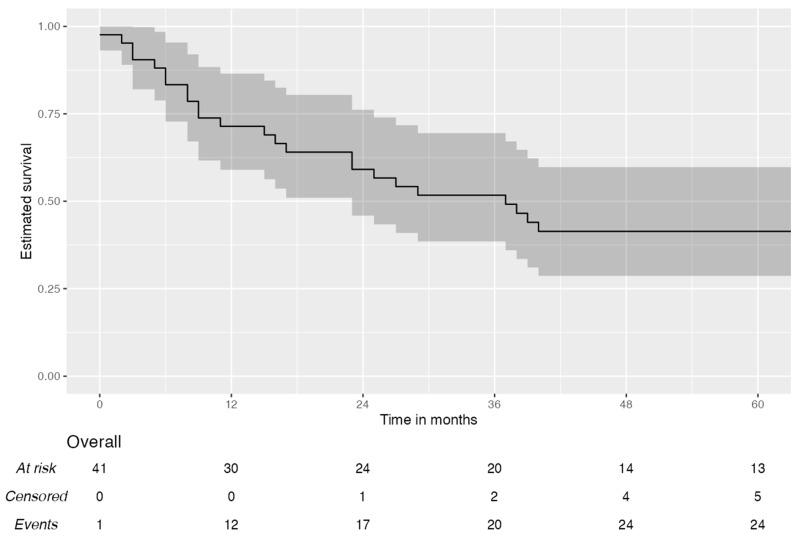
Overall survival curve.

Figure 2 shows the disease-free survival (DFS) curve. The median DFS was 38 months, while the 1–3 and 5-year disease-free survival was, respectively, 72% [95% CI, 60–87.8%], 62% [95% CI, 48–80.6%], and 35% [95% CI, 20–60.4%]. Figure 3 reports on the overall survival (OS) of the patients; the median value was 37 months, while the 1–3 and 5-year OS was 71% [95% CI; 59–86.5%], 52% [95% CI, 38.5–69.5%], and 41% [95% CI, 28.7–59.8%], respectively.

Univariate analysis showed that wedge resection (HR 16.96, *p* = 0.018) and preoperative COPD (HR 11.73, *p* = 0.005) were significantly associated with a higher risk of recurrence at follow-up, while vascular infiltration (HR 3.33, *p* = 0.023), wedge resection (HR 15.94, *p* = 0.018), and a high Charlson Comorbidity Index (CCI) (HR 1.32, HR = 0.038) were linked to increased mortality risk. Conversely, the female sex appeared to have a protective role (HR 0.34, *p* = 0.031). Multivariable analysis identified vascular and/or vertebral infiltration as a significant predictor of recurrence (HR 3.72, *p* = 0.017) and mortality (HR 3.27, *p* = 0.010), whereas pathological complete response reduced the rate of recurrence (HR 0.18, *p* = 0.027) and mortality risk (HR 0.33, *p* = 0.018). Additionally, a higher CCI was a predictor of worse survival (HR 1.48, *p* = 0.006) (Table 3 and Table 4).

## 4. Discussion

The treatment of Pancoast tumors has evolved over the years. Nowadays, the standard of care consists of induction chemoradiotherapy, which has been shown in several trials [5,6] to be superior to radiotherapy alone. Although our center (Thoracic Surgery Unit, University Hospital of Padua, Italy) began its surgical experience of Pancoast tumors more than 40 years ago, we intentionally chose to analyze our more recent experience with multimodality treatment to ensure a more homogenous patient population. The initial cases were managed using preoperative irradiation, followed by en bloc tumor resection involving the chest wall. Radiotherapy served a dual purpose: primarily for pain relief and, secondarily, with a cytoreductive intent. Following the encouraging outcomes from the Southwest Oncology Group (SWOG 9416) and Japan Clinical Oncology Group (JCOG 9806) trials [5,6], the treatment approach evolved in the 1990s from a bimodality to a trimodality regimen. Over time, we have also refined our chemoradiotherapy protocols. In particular, modifications were made to the radiotherapy component, as specified in the methods section. In light of these changes, we intentionally chose to analyze our more recent experience with multimodality treatment.

All the patients involved in our study received a neoadjuvant treatment, with the majority (*n* = 34, 79%) undergoing induction chemoradiotherapy. The use of trimodal therapy proved to be both feasible and effective, as no patients discontinued treatment due to toxicity, and no disease progression was reported during or after treatment. Moreover, there were no delays to surgery and a significant proportion of our patients (65%, *n* = 28) experienced either an improvement or complete resolution of local pain after neoadjuvant treatment. Interestingly, in one recent case, immunotherapy was administered in addition to the standard protocol, and the patient achieved a pathological complete response. Although data on immunotherapy specifically for Pancoast tumors remain limited [9,10], its combination with chemotherapy in a neoadjuvant setting has already been shown to improve event-free survival and increase the rate of pathological complete response [12] in NSCLCs. Given these promising results, future treatment strategies should include anti-PD1/PD-L1 blockade therapy, alongside chemoradiotherapy, specifically for Pancoast tumors.

Concerning the surgical procedure, the primary surgical approach involved a Shaw–Paulson thoracotomy (42 patients, 98%), which was also preferred in the five cases with vascular infiltration. It consists of an extended posterolateral thoracotomy, with the incision starting anteriorly, extending posteriorly around the tip of the scapula, and continuing vertically up to C7. The pleural cavity is typically accessed through the fourth intercostal space, and a scapular retractor is used to elevate the scapula.

Based on our surgical experience, posterolateral thoracotomy remains the optimal approach not only for posterior Pancoast tumors, but also in the case of subclavian vessel involvement. This technique provides excellent exposure of the thoracic inlet region, facilitating vascular resections and reconstructions when necessary. Additionally, it is the most effective approach for safely performing rib excisions and lung resections. With advancements in minimally invasive surgery, hybrid techniques can be considered in selected cases. Ultimately, no single approach is universally superior; the choice should be tailored to the surgeon’s expertise and the tumor’s characteristics.

In our cohort, anatomical parenchymal resection was performed in 42 patients (98%), with upper lobectomies being the most common procedure. In only one patient, a wedge resection with wide surgical margins was necessary due to the patient’s poor respiratory function and cardiovascular comorbidities. This type of resection was associated with a higher risk of recurrence and mortality in the univariate analysis. This may be explained by the fact that it is a non-anatomical resection and by the poor performance status of the patients, who required a limited parenchymal resection to preserve their lung function. We believe that emphasizing the importance of anatomical parenchymal resections and accurate patient selection is essential to ensure an oncologically sound surgical approach is followed.

Surgery for Pancoast tumors is highly complex and invasive, with significant morbidity and mortality rates associated with surgical resection of around 10–55% and 0–9%, respectively, reported in previous studies [13,14,15,16,17,18,19]. In our study, the rate of post-operative complications was low, with only seven patients (16%) experiencing at least one post-operative complication. The 30- and 90-day mortality rates were 0% and 6.9%, respectively. One of the main risks associated with multimodal treatment is the onset of treatment-related complications and a high morbidity rate, which may lead to treatment discontinuation and disease progression. Therefore, careful and thorough patient selection is essential. Patients undergoing multimodal treatment must be in good overall condition, with adequate respiratory and cardiovascular reserves. When necessary, second-level examinations, such as cardiopulmonary stress testing or lung scintigraphy, can aid proper patient selection. These assessments should be performed in cases of uncertainty both before and after medical treatment, as part of the preoperative evaluation.

The low rate of complications and peri-operative mortality reported in our case series highlights not only the importance of properly selecting suitable surgical candidates, but also the necessity of performing surgery for Pancoast tumors at high-volume centers with a dedicated multidisciplinary team. Moreover, multivariable analysis revealed a significant association between a high Charlson Comorbidity Index (CCI) and an increased mortality risk (HR 1.48, HR = 0.006), further underscoring the importance of careful patient selection.

A key factor in Pancoast surgery is the radicality of the resection. In our series, a radical (R0) resection was achieved in the majority of patients (36 patients, 84%), consistent with previous reports [20]. Among the seven patients with uncomplete resection (R+), three had brachial plexus involvement. In these cases, incomplete resection was primarily due to the challenge of obtaining clear surgical margins on the neural structures. Moreover, we would like to emphasize that our population comprised 39 patients (91%) with a disease stage higher than IIIA. Despite this, multivariable analysis, in our case series, revealed that the radicality of resection (R0 vs. R+) was not an independent predictor of recurrence risk (HR = 1.21, *p* = 0.778).

The rate of pathological complete response (PCR) in our population (23%) is consistent with that in previously reported studies [20] and further supports the effectiveness of multimodal therapy. The higher PCR rate reported by other authors [21,22] may be largely attributed to the inclusion of patients with lower disease stages compared to our cohort. Furthermore, we observed a direct effect of PCR on patients’ survival; in fact, a multivariate analysis of the patients with PCR found that they had a statistically significant difference in terms of their survival (*p* = 0.018) and a lower probability of recurrence (*p* = 0.027) compared to all the other patients.

Despite the aggressiveness and locally advanced nature of this subset of NSCLCs, we reported satisfactory medium- and long-term survival outcomes, with overall 1-, 3-, and 5-year survival rates of 71%, 52%, and 41%, respectively. Our results align with the average outcomes reported in other studies [18,19,20,21,22]. A review of the literature reveals variability in the overall 5-year survival rates, which, in our opinion, can largely be attributed to the heterogeneity of the patient population and the variations in the induction treatment regimens applied over the years.

Our study has some limitations primarily due to its retrospective nature. Additionally, although we attempted to analyze our more recent experience to minimize selection bias, variations in the induction chemoradiotherapy protocols applied over time may have resulted in a heterogenous patient population.

## 5. Conclusions

Pancoast tumor treatment is complex and should be performed at high-volume centers with a dedicated multidisciplinary team. Currently, trimodal therapy composed of induction chemoradiotherapy, followed by surgery, offers the best short- and long-term outcomes. However, survival is still not satisfactory, with high relapse rates.

Future treatment strategies will likely incorporate tyrosine kinase inhibitors and anti-PD1/PD-L1 therapies, alongside existing chemoradiation regimes, which are becoming the standard of care for all other NSCLCs, even in the neoadjuvant setting. As already observed [10], this combination may lead to a higher PCR rate in regard to Pancoast tumors, and, hopefully, to better distant control of the disease.

## Figures and Tables

**Table 1:** General characteristics of the overall study population.

Variables	*n* = 43
**Gender**	
M	33 (77%)
F	10 (23%)
**Age (y)**	63 (57–78)
**Smoking habit**	
Yes/former smoker	40 (93%)
No	3 (7%)
**Comorbidities**	
Arterial hypertension	13 (30%)
Cardiac disease	5 (12%)
COPD	3 (7%)
Diabetes	4 (9%)
**CCI**	3 (2–3)
**Pre-treatment pain**	
Yes	39 (91%)
No	4 (9%)
**Pain distribution**	
Shoulder	14 (32%)
Shoulder–arm	19 (44%)
Shoulder–arm–hand	6 (14%)
**Claude Bernard-Horner Syndrome**	
Yes	6 (14%)
No	37 (86%)
**Side of the disease**	
Right	24 (56%)
Left	19 (44%)
**Diagnostic procedure**	
CT guided	30 (70%)
Bronchoscopy	10 (23%)
Other	3 (7%)
**Clinical Stage (TNM 8th edition)**	
IIB	4 (9%)
IIIA	22 (51%)
IIIB	13 (31%)
IIIC	3 (7%)
IV	1 (2%)

COPD: chronic obstructive pulmonary disease; CCI: Charlson Comorbidity Index, CT: computed tomography. Data are shown as absolute numbers (percentages) for categorical variables and as the median (interquartile range) for continuous variables.

**Table 2:** Data on the patients’ multimodal treatment.

Variables	*n* = 43
**Vertebral infiltration**	
Yes	4 (9%)
No	39 (91%)
**Vascular infiltration**	
Yes	5 (12%)
No	38 (88%)
**Surgical resection**	
Lobectomy	36 (84%)
Segmentectomy	2 (5%)
Wedge	1 (2%)
Bilobectomy	2 (5%)
Pneumonectomy	1 (2%)
Other	1 (2%)
**Surgical approach**	
Posterior (Shaw–Paulson)	42 (98%)
Combined (VATS + anterior thoracotomy)	1 (2%)
**Chest wall resection**	
One rib	4 (9%)
Two ribs	11 (26%)
Three ribs	23 (53%)
Four ribs	5 (12%)
**Post-operative complications (Clavien–Dindo)**	
1	29 (67%)
2	12 (28%)
3	2 (5%)
**Completeness of resection**	
R0	36 (84%)
R+	7 (16%)
**Induction therapy**	
CT + RT	34 (79%)
RT	3 (7%)
CT	5 (12%)
CT + RT + immunotherapy	1 (2%)
**Preoperative histology**	
Adenocarcinoma	26 (60%)
Squamous	12 (28%)
Large cell carcinoma	1 (2%)
Other	4 (10%)
**Pathological stage (TNM 8th edition)**	
Pathological complete response	10 (23%)
IB	1 (2%)
IIB	3 (7%)
IIIA	23 (53%)
IIIB	1 (2%)
IIIC	4 (9%)
IV	1 (2%)
**Status at follow-up**	
Alive	10 (23%)
Dead	33 (77%)
**Recurrence at follow-up**	
No	23 (53%)
Local	13 (30%)
Distant	5 (12%)
Local + distant	2 (5%)

CT: chemotherapy; RT: radiotherapy. Data are shown as absolute numbers (percentages) for categorical variables.

**Table 3:** Multivariable analysis for recurrence.

Variable	HR (95%CI)	*p* Value
**Vascular and/or vertebral infiltration**		0.017
No	-	
Yes	3.72 (1.26–11)	
**Pathological complete response**		0.027
No	-	
Yes	0.18 (0.04–0.83)	
**Completeness of resection**		0.778
R0	-	
R+	1.21 (0.33–4.42)	
**Neoadjuvant regimens**		0.476
Other	-	
Neoadjuvant CT + RT	0.66 (0.21–2.07)	

**Table 4:** Multivariable analysis for survival.

Variable	HR (95%CI)	*p* Value
**Vascular and/or vertebral infiltration**		0.01
No	-	
Yes	3.27 (1.33–8.07)	
**Pathological complete response**		0.018
No	-	
Yes	0.33 (0.13–0.83)	
**Charlson Comorbidity Index (CCI)**	1.48 (1.12–1.95)	0.006
**Neoadjuvant regimens**		0.815
Other	-	
Neoadjuvant CT + RT	0.90 (0.35–2.26)	

## Data Availability

The data presented in this study are available on request from the corresponding author due to privacy.
